# Pulmonary Pleomorphic Carcinoma With Mesenchymal-Epithelial Transition Factor (MET) Mutation: An Aggressive Entity

**DOI:** 10.7759/cureus.92752

**Published:** 2025-09-19

**Authors:** Yuta Takemura, Kazuki Fukumoto, Akinori Sasaki

**Affiliations:** 1 Internal Medicine, Tokyo Bay Urayasu Ichikawa Medical Center, Urayasu, JPN; 2 Gastroenterology, Tokyo Bay Urayasu Ichikawa Medical Center, Urayasu, JPN

**Keywords:** comprehensive genomic testing, met exon 14 skipping mutation, met inhibitor, non-small cell lung cancer, pulmonary pleomorphic carcinoma

## Abstract

Pulmonary pleomorphic carcinoma (PPC) is a rare, aggressive non-small cell lung cancer (NSCLC) subtype characterized by rapid progression and challenging diagnosis from a limited biopsy. These challenges delay treatment initiation. Comprehensive genomic panel profiling helps identify actionable driver mutations, such as mesenchymal-epithelial transition factor (MET) exon 14 skipping mutations, enabling molecular targeted therapy planning. We present a case of an older female patient with suspected NSCLC and rapid clinical deterioration, where biopsy did not confirm the specific subtype. Genotype testing identified a MET exon 14 skipping mutation. A MET inhibitor treatment was planned; however, the patient died before treatment began. Postmortem autopsy confirmed the diagnosis of PPC. This case highlights the importance of early comprehensive genomic profiling in suspected NSCLC with inconclusive histology based on a fine-needle biopsy (FNB) to facilitate timely and potentially beneficial therapeutic planning in rapidly progressing malignancies such as PPC.

## Introduction

Lung cancer is the most common cancer type and the leading cause of cancer-related mortality worldwide [1]. Non-small cell lung cancer (NSCLC) is the most common lung cancer subtype, with 60%-70% diagnosed at advanced stage Ⅳ. Although various chemotherapy regimens exist for patients with advanced NSLC, prognosis remains poor, with a median survival of approximately 1.5 years [2]. However, a paradigm shift in NSCLC treatment occurred with the identification and targeted treatment of genomic oncogenic drivers such as epidermal growth factor receptor (EGFR), anaplastic lymphoma kinase (ALK), and mesenchymal-epithelial transition factor (MET) exon 14 skipping. Patients with NSCLC with driver gene mutations have better prognoses than those without [2]. Presently, targetable driver genes are identified in approximately 60% of lung adenocarcinoma cases in Western populations and up to 80% in Asian populations [3].

Adenocarcinoma is the most common NSCLC histological subtype, followed by squamous cell carcinoma [4]. Pulmonary pleomorphic carcinoma (PPC) is a rare NSCLC subtype, defined as a malignancy that contains at least 10% spindle or giant cell components, accounting for approximately 0.1% of all NSCLC cases [5]. PPC progresses rapidly and can deteriorate the patient’s condition before treatment can begin [6,7]. Treatable driver mutations such as EGFR, ALK, and MET exon 14 skipping have been detected in patients with PPC [6,8]. Therefore, patients with PPC have a poor prognosis; however, their prognosis may improve depending on the results of driver gene testing.

PPC can harbor actionable oncogenic driver gene mutations such as EGFR, ALK, B-Raf proto-oncogene, serine/threonine kinase (BRAF), Kirsten rat sarcoma viral oncogene homolog (KRAS), and MET exon 14 skipping. In this context, “driver mutations” are key genetic changes that fuel a tumor’s growth. The process of searching for these alterations is called “comprehensive genomic testing,” a laboratory analysis of a tumor’s DNA. This testing is essential because finding a specific driver mutation can guide treatment decisions, enabling the use of targeted therapies that are designed to selectively block the effects of that particular mutation.

Among these actionable mutations, MET exon 14 skipping is a key oncogenic driver found in approximately 3%-4% of NSCLC cases [9]. The MET proto-oncogene encodes a receptor tyrosine kinase, and aberrant activation via this exon 14 skipping mutation leads to downstream signaling that promotes tumor cell proliferation, survival, and invasion [10]. Potent MET tyrosine kinase inhibitors have been developed to counter this alteration. Gumarontinib, a selective MET inhibitor used in this case, was approved in Japan in 2024 for the treatment of advanced or recurrent NSCLC with MET exon 14 skipping, based on its demonstrated clinical efficacy [11]. The identification of such therapeutic targets is facilitated by comprehensive genomic panels such as the Oncomine™ Dx Target Test. This next-generation sequencing (NGS)-based companion diagnostic simultaneously analyzes both DNA and RNA from a single tumor sample to detect multiple biomarkers. For NSCLC, its panel is designed to identify critical mutations in genes such as EGFR, BRAF, and KRAS; fusions in ALK, proto-oncogene 1, receptor tyrosine kinase (ROS1), and rearranged during transfection (RET); and alterations such as MET exon 14 skipping, thereby enabling the selection of appropriate molecular targeted therapies [12].

This case report describes a patient whose PPC was confirmed postmortem, and the process of diagnosis and management particularities will be discussed.

## Case presentation

**Figure 1:**
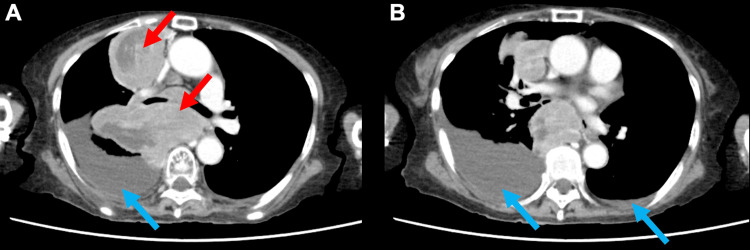
Initial chest CT image showing a bulky mediastinal tumor and bilateral pleural effusions Figure 1A shows a bulky mediastinal tumor (red arrows) compressing the esophagus. Figure 1A and Figure 1B demonstrate bilateral pleural effusions, which are more predominant on the right side (blue arrows). CT: computed tomography

**Figure 2:**
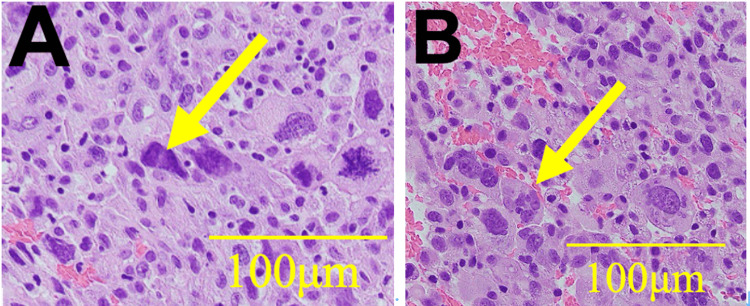
Histopathology of the tumor specimen obtained via endoscopic ultrasound-guided fine-needle biopsy of the mediastinal mass Figure 2A and Figure 2B show different sites from the same tumor specimen. Histopathological examination revealed large nuclei (yellow arrows in Figure 2A) and multinucleated cells (yellow arrow in Figure 2B). A subset of tumor cells exhibited increased nuclear chromatin, irregular cytoplasmic morphology resembling squamous cell features, and eccentric nuclei. However, a definitive histological subtype could not be determined, and the findings were suggestive of non-small cell carcinoma.

An older female patient in her early 80s presented with fatigue and loss of appetite persisting for several months and was subsequently admitted to our hospital. She had no history of smoking and a medical history of poliomyelitis and cataracts. Prior to symptom onset, her Eastern Cooperative Oncology Group (ECOG) Performance Status (PS) deteriorated from 1 (fully ambulatory and able to carry on light work) to 4 (completely disabled and confined to bed) before admission [13]. Computed tomography (CT) revealed a large anterior mediastinal mass compressing the esophagus and bilateral pleural effusions (Figure 1). However, CT did not show significant metastases to the bone, adrenal glands, or liver. Furthermore, a brain magnetic resonance imaging (MRI) revealed no evidence of metastases. Tumor markers were elevated: pro-gastrin-releasing peptide (ProGRP), 48.8 pg/mL; neuron-specific enolase (NSE), 37 ng/mL; Sialyl-Lewis X (SLX), 52.1 U/mL; cytokeratin 19 fragment (CYFRA), 7.1 ng/mL; and interleukin-2 receptor (IL-2R), 3,532 U/mL (Table 1). Based on findings from the CT scan and tumor markers, small cell lung cancer, NSCLC, and malignant lymphoma were considered as differential diagnoses. Subsequently, an endoscopic ultrasound-guided fine-needle biopsy (EUS-FNB) of the mediastinal lesion was performed, using a 19-gauge needle to obtain adequate tissue for both histological and genomic analysis (Figure 2). Histopathological examination of the tumor biopsy sample indicated NSCLC, although the specific histological subtype could not be determined. Furthermore, an immunohistochemical panel was performed, but the results were non-contributory for definitive subtyping.

**Table 1:** Laboratory findings of tumor markers ProGRP: pro-gastrin-releasing peptide, NSE: neuron-specific enolase, SLX: Sialyl-Lewis X, CYFRA: cytokeratin 19 fragment, IL-2R: interleukin-2 receptor

Tumor marker	Measured value	Normal range
ProGRP	48.8 pg/mL	<81 pg/mL
NSE	37 ng/mL	<16.3 ng/mL
SLX	52.1 U/mL	<38 U/mL
CYFRA	7.1 ng/mL	<3.5 ng/mL
IL-2R	3,532 U/mL	122-613 U/mL

Given the patient’s advanced stage and exceptionally rapid clinical decline, the immediate clinical priority was to identify a potential molecular target for therapy, rather than to pursue a more definitive histological classification via further invasive procedures. To identify potentially actionable genetic alterations, next-generation sequencing (Oncomine™ Dx Target Test) was performed and revealed a MET exon 14 skipping mutation. Based on the evidence of invasion into the esophagus (T4) and the presence of pleural effusion, the disease was classified as stage IV. Consequently, the initiation of treatment with the MET inhibitor gumarontinib was planned [8]. However, due to a severe loss of appetite and the onset of terminal delirium, oral administration of gumarontinib was not feasible. Thus, she succumbed to tumor progression on hospital day 25, before gumarontinib administration (Table 2). A postmortem pathological autopsy was subsequently performed, revealing an approximately 10 cm tumor in the right upper lung lobe. This postmortem histopathological evaluation revealed that the tumor predominantly consisted of spindle and giant cells, with poorly differentiated adenocarcinoma components in part. Based on these findings, the tumor was eventually diagnosed as PPC.

**Table 2:** Timeline of key clinical events CT: computed tomography, EUS-FNB: endoscopic ultrasound with fine-needle biopsy, IVC: inferior vena cava, CMO: comfort measures only

Hospital day	Key clinical events and interventions
Day 1	Admitted to the hospital. Contrast-enhanced CT reveals a large mediastinal tumor. EUS-FNB is performed to obtain a specimen.
Day 4	Initial histopathology report of the EUS-FNB specimen suggests a malignant tumor.
Day 11	Comprehensive genomic profiling identifies a MET exon 14 skipping mutation.
Day 20	A large right pleural effusion and a collapsed IVC.
Day 21	Quetiapine is initiated for terminal delirium.
Day 22	The planned MET inhibitor (gumarontinib) arrives, but oral administration is not feasible due to the patient’s deteriorating condition. Dexamethasone is initiated.
Day 23	Dexamethasone is discontinued, and a 0.5 mg fentanyl patch is applied for pain management.
Day 24	The fentanyl patch is replaced with intravenous morphine. The decision is made to transition to CMO.
Day 25	The patient died.

## Discussion

This case of a large mediastinal tumor provided key insights into the diagnostic and therapeutic process. First, in a suspected case of NSCLC with an indeterminate histological subtype based on biopsy pathology, submission of a comprehensive cancer gene panel, while considering the possibility of rare malignancies such as PPC, enabled the successful identification of a MET exon 14 skipping mutation. Second, even without a definitive pathological diagnosis, the gene panel allowed the construction of a treatment plan, incorporating the potential use of a molecular targeted therapy.

PPC is typically diagnosed at a median age of approximately 66, and it demonstrates a higher prevalence in men (58%-76%), with a strong correlation to a history of smoking [14,15]. In addition, the lesions most frequently affect the upper lobes, with a predilection for the right lung [14]. In this case, the patient was an older female non-smoker, deviating from established epidemiological trends. The tumor progressed rapidly and was located in the right upper lobe, aligning with known clinical PPC features. In addition, histologically, PPC is defined by the presence of both epithelial and sarcomatoid components, including spindle and giant cells. The epithelial component of PPC may include adenocarcinoma, squamous cell carcinoma, or large cell carcinoma elements [16]. The sarcomatoid component consists of undifferentiated elements and is associated with a high degree of biological aggressiveness [17]. A definitive diagnosis typically requires comprehensive histopathological examination of resected specimens, as biopsy samples alone are generally insufficient for diagnosis, making early detection challenging [18]. In this case, histopathology revealed undifferentiated NSCLC characterized by prominent spindle and giant cell components. The strong presence of sarcomatoid features likely indicated a high tumor grade and contributed to the tumor’s aggressive clinical behavior.

PPC may harbor various oncogenic driver mutations, allowing for early development of molecular targeted treatment strategies, even before definitive histopathological diagnosis. PPC harbors various actionable oncogenic driver gene mutations, including KRAS (27%), EGFR (8%), and PIK3CA (7%). Among these, MET exon 14 skipping mutations are present in approximately 8% of PPC cases [8]. Although evidence remains limited to individual case reports, several have described marked clinical responses to MET inhibitors in patients with PPC harboring MET exon 14 skipping mutation [17]. Ongoing investigations are evaluating the therapeutic efficacy of MET-targeted agents in this molecular subset [19,20]. PPC is a rare and aggressive malignancy where definitive diagnosis from small biopsy samples is often challenging. However, since PPC can harbor actionable driver mutations, early comprehensive genomic profiling is critical. In this clinical context, identifying a targetable mutation by genomic profiling can be of immediate therapeutic importance as it directly guides the use of molecular targeted therapies. In this case, MET exon 14 skipping was identified via comprehensive genomic profiling before a definitive diagnosis was made. This early gene mutation identification enabled the planning of MET-targeted therapy. Notably, several case reports have described therapeutic responses, commonly referred to as the “Lazarus effect,” in patients with MET exon 14 skipping-positive NSCLC and poor PS following MET inhibitor treatment [21-23]. Although MET inhibitor therapy with gumarontinib was not initiated prior to the patient’s death in this case, previous reports support its potential for therapeutic benefit.

Finally, we acknowledge some limitations in this report. Our report focused solely on the identified MET exon 14 skipping mutation as the primary therapeutic target. The full data from the comprehensive genomic panel, including the status of potential co-mutations, which could influence prognosis or therapeutic response, were not available for this case report.

## Conclusions

In conclusion, PPC is a rare, rapidly progressing malignancy with a poor prognosis, primarily owing to the challenges in achieving a definitive diagnosis. When a mediastinal mass demonstrates heterogeneous histological features on biopsy, making classification and diagnosis challenging, comprehensive genomic profiling should be considered. Early identification of actionable driver mutations through such profiling may enable the timely initiation of personalized therapy, including molecular targeted therapies, which is essential for improving patient outcomes. This case, therefore, underscores the need to consider advocating for standardized protocols that incorporate early comprehensive genomic profiling in patients with suspected high-grade or rapidly progressing NSCLC, even when histology is inconclusive. As recent studies have suggested, the timing of genomic profiling matters, and earlier testing in patients with lung cancer is associated with improved survival outcomes. Establishing such protocols could be a critical step in optimizing treatment pathways and ensuring that patients have the best possible chance to benefit from precision medicine.
